# Intravenous methadone in the management of acute postoperative pain in a chronic cancer pain patient: A case report and review of the literature

**DOI:** 10.1002/ccr3.8332

**Published:** 2023-12-13

**Authors:** Khang Duy Ricky Le, Jean Hua

**Affiliations:** ^1^ Department of General Surgical Specialties The Royal Melbourne Hospital Melbourne Victoria Australia; ^2^ Department of Surgical Oncology Peter MacCallum Cancer Centre Melbourne Victoria Australia; ^3^ Geelong Clinical School Deakin University Geelong Victoria Australia; ^4^ Department of Medical Education Melbourne Medical School The University of Melbourne Melbourne Victoria Australia; ^5^ Faculty of Pharmacy and Pharmaceutical Sciences Monash University Parkville Victoria Australia; ^6^ Department of Pharmacy The Royal Melbourne Hospital Melbourne Victoria Australia

**Keywords:** chronic pain, methadone, postoperative pain management, surgical oncology

## Abstract

**Key Clinical Message:**

The current landscape of literature highlights that there is insufficient well‐powered and robust evidence to support the integration of intravenous methadone into current guidelines and frameworks in supporting the pain management of cancer patient with complex pain syndromes. However, there is preliminary evidence, both from the literature as well as this case study that highlights intravenous methadone may be efficaciously and safety used for the management of postoperative pain in cancer patients with chronic pain undergoing operative management. Further research is required to fully elucidate key considerations of integrating this medication into clinical practice including consideration into dosing, opioid conversion, tolerance, and safety.

**Abstract:**

Methadone is a broad‐spectrum analgesic with long duration of effect. Its multimodal mechanism of action, such as through effects on mu‐opioid receptor and presynaptic N‐methyl‐D‐aspartate receptors, has led to its current use in the management of opioid dependence in the community and in palliative care. These properties however make methadone appealing in the management of postoperative pain, particularly for patients with complex analgesic requirements. We report on an interesting case whereby intravenous methadone was effectively used for postoperative analgesia in a 56‐year‐old female with complex chronic pain secondary to a mucinous pelvic neoplasm of unclear primary who underwent palliative resection. Further, we review the literature surrounding usage of methadone in this setting to understand current challenges and barriers to implementation of methadone as an analgesia option for chronic pain patients following surgery. To do this, a case report and literature review was conducted in accordance to the CARE case report guidelines. The patient provided written consent for the de‐identification and use of their medical information and data for the generation and publication of this case report. Our case report and literature review demonstrate there remains significant heterogeneity, unfamiliarity, and scarce use of intravenous methadone in the perioperative and postoperative space in the management of patients with complex pain regimens such as chronic cancer pain patients. Despite this, our case report and literature review highlight as a broad analgesic, intravenous methadone warrants consideration following more rigorous research and development of safe use guidelines into its use for this purpose.

## Graphical abstract

Timeline to peri‐operative methadone use for analgesia for a cancer patient with chronic pain undergoing palliative surgery.

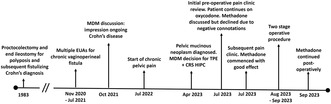

## 1 BACKGROUND

Methadone is an opioid analgesic with potent mu‐opioid agonist activity.^1^, ^2^ It is also an inhibitor of monoamine re‐uptake and presynaptic N‐methyl‐D‐aspartate (NMDA) receptors.^1^, ^2^ In doing so, methadone provides adequate analgesia through its broad‐spectrum potent opioid activity and reinforcement of descending inhibitory pathways, endogenous pain modulation, and inhibitor of the “wind‐up” phenomenon and central sensitization.^3^, ^4^ In addition, methadone exhibits a long duration of effect which has seen its use for the management of chronic opioid dependence in the community. These pathways additionally have been demonstrated to be effective in the treatment of acute and chronic cancer pain, with a growing number of studies reporting on their use for this purpose, in some cases even as the first‐line agent.^5^, ^6^

There has been consideration of the potential for methadone as an analgesic within the postoperative period, particularly for patients with complex analgesia requirements.^7^ Despite this, methadone is highly regulated, with no current evidence base to inform the development of appropriate guidelines and frameworks to guide its use outside that of opioid replacement therapy and leaves methadone an underused medication in pain medicine. Further barriers to its wider use can be attributed to unfamiliarity with the drug and a lack of consensus opinion on dose conversions from comparator opioids. Nonetheless, it is known that intravenous (IV) methadone accelerates achievement of therapeutic plasma concentrations and can therefore offer a potential short‐onset and long‐offset analgesia. This is highly appealing, particularly for cancer patients with chronic pain undergoing oncological surgery whereby there may be periods in which pain may be poorly controlled and oral routes are unavailable.

Herein, we report on a case in which IV methadone was utilized for efficacious postoperative pain relief in a patient with chronic pain secondary to malignancy. Further, we explore the current and relevant literature and frameworks evaluating the use of IV methadone in postoperative pain management.

## 2 CASE PRESENTATION

**FIGURE 1:**
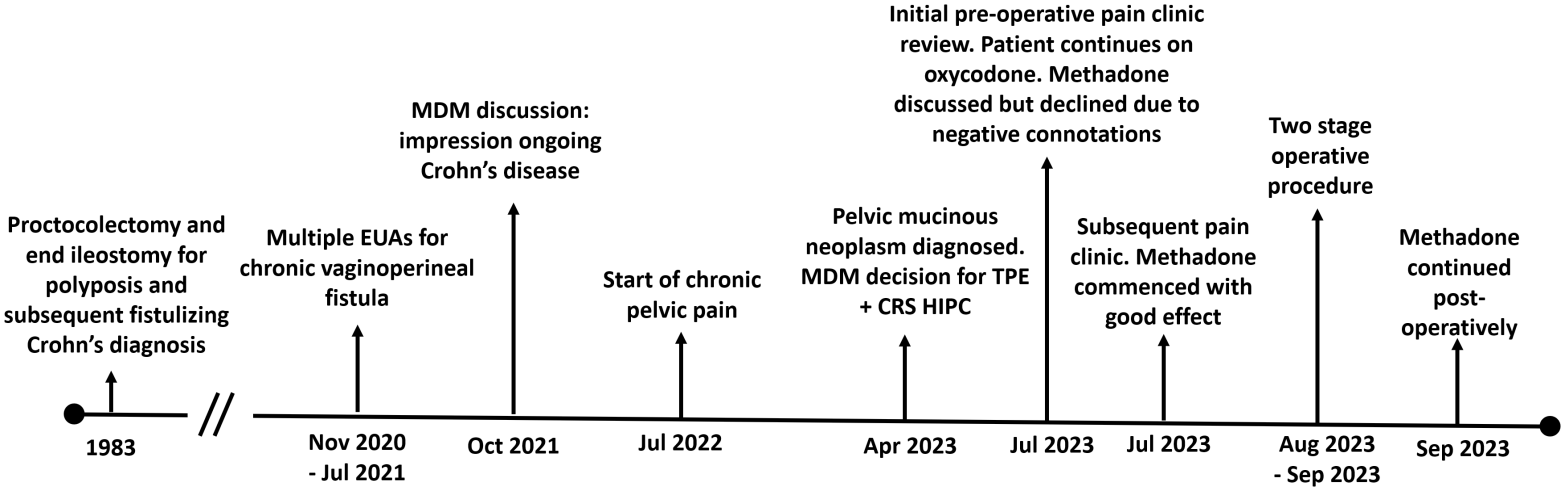
Timeline of events.

A brief overview of events is summarized in the timeline depicted by Figure 1.

### 2.1 Events leading to hospital admission

A 56‐year‐old female was pre‐emptively referred to the acute and persistent pain service 7 weeks prior to palliative oncological surgery for optimization of persisting pelvic pain. The planned operative procedure includes a total pelvic exenteration (TPE) with concurrent cytoreductive surgery and hyperthermic intraperitoneal chemotherapy (CRS HIPEC) for mucinous pelvic neoplasm of unclear primary.

### 2.2 Medical background of patient

The patient has complex gastrointestinal medical and surgical history. The patient first underwent a proctocolectomy and end ileostomy formation at age 16 for juvenile polyposis and was then subsequently diagnosed with fistulizing perianal Crohn's disease in which over her lifetime has been treated with multiple immunomodulatory and immunosuppressive medications including azathioprine, ustekinumab, adalimumab, and infliximab. At the time of review, the patient was no longer receiving any maintenance therapy. Since 2020, the patient had undergone multiple examinations under anesthesia (EUA) for chronic vaginoperineal fistula with seton insertion. Specifically, the patient had a chronic pelvic mucinous cyst with discharge into the perineum and posterior left thigh. These occurred in early November 2020, mid November 2020, mid‐ to late June 2021. Histopathology specimens from these demonstrated non‐specific inflammation and were all benign. These admissions were complicated by perianal sepsis requiring multiple debridements and IV antibiotic therapy. The patient's case was discussed in a specialist multidisciplinary discussion around mid October 2021. During this discussion, a magnetic resonance image (MRI) from June 2021 of the pelvis that demonstrated no definitive evidence of malignancy, a computerized tomography (CT) of the abdomen/pelvis from July 2021 that demonstrated interval reduction in size of the known multiloculated trans‐spatial pelvic collection, and a positron emission tomography (PET) scan from October 2021 that demonstrated no FDG‐avid disease was reviewed. The consensus was that the imaging and pathology were indicative of Crohn's disease process.

The patient was re‐referred to operative service in late April 2023 for a progressive pelvic mucinous mass with new ureteric compression and ongoing mucinous discharge from natal cleft and posterior left thigh. The patient proceeded to a repeat EUA of the anorectal region with subsequent pathology identifying a low‐grade mucinous neoplasm of unclear primary. After a further specialist multidisciplinary meeting and shared decision‐making with the patient, a decision was made to proceed to TPE, CRS HIPEC, and ileal conduit as a palliative procedure to improve pelvic pain, mucinous leakage, and quality of life for the patient.

### 2.3 Presurgical pain history

The patient presented to the acute and persistent pain clinic approximately 7 weeks before her scheduled operation. At this time, the patient's pain history was clarified.

She reported 12 months of persistent pelvic pain presumably secondary to the pelvic mass. The pain was described as a pressure‐like sensation which upon bearing down would change to a constant aching sensation. The patient also reported a burning‐type pain in the pelvis associated with urinary flow. There were no other typical neuropathic pain descriptors associated with any other areas. The patient reported her pain was exacerbated by multiple factors including urinary frequency (5–6 times per day on average) and performing usual activities of daily living. The pain limited the patient's ability to walk to a duration of only approximately 4–5 min and her tolerance for standing still was reduced to only 10 min at a time. Temporary reprieve was found only in the sitting position. Reports of anhedonia and low mood were largely attributed to pain with poor sleep also a factor.

From a social perspective, the patient lives with her mother who is unwell with high care needs but is reasonably independent with personal activities of daily living. The patient has been financially dependent on a disability pension since the age of 16. She does not consume alcohol or smoke and has an allergy to penicillin (anaphylaxis). She rarely leaves the house due to pain.

### 2.4 Previous pain management

At the time of her pain clinic review in July 2023, the patient was on an analgesia regimen consisting of oxycodone liquid as required (up to 40 mg per day) with additional temazepam 10 mg as needed for sleep. She was also prescribed 45 mg of mirtazapine at night. She had previously tried sustained‐release (SR) oxycodone‐naloxone as well as tapentadol SR and transdermal buprenorphine with poor effect. It was suspected that the low efficacy of oral formulations was due to poor absorption in the setting of ileostomy. Additionally, the patient did not tolerate buprenorphine which had caused a peri‐stomal rash. Methadone was discussed as an additional line of analgesia at this time; however, the patient opted to revisit this discussion at a later time given the negative connotations associated with the drug. She continued with her immediate‐release (IR) oxycodone in the interim.

The patient re‐presented to the pain clinic 2 weeks following her review ongoing persistence of the aforementioned pelvic pain. She had now been taking up to 50 mg of oxycodone per day. Through a shared decision‐making process, the patient agreed to commence oral methadone at a dose of 5 mg twice a day with IR oxycodone to be used as a bridge for symptom control. Over the subsequent weeks leading up to her surgery date, the patient uptitrated methadone to an oral dose of 5 mg in the morning and 10 mg at night which allowed her symptoms to become more manageable. She had downtitrated her oxycodone liquid dose at the same time.

### 2.5 Surgical admission and postoperative pain management

The patient proceeded to surgery with a planned two‐stage procedure. The first stage occurred on first day of admission where the patient underwent an open completion posterior exenteration with hysterectomy including resection of lateral and posterior wall of the vagina, bilateral oophorectomy, left nephrectomy and let adrenalectomy, insertion of bilateral ureteric catheters, and temporary closure of the abdomen. Postoperatively the patient was routinely managed in the intensive care unit (ICU) with analgesia regimen consisting of a fentanyl infusion (200mcg/h), IV paracetamol 1 g six hourly and breakthrough 5 mg IV methadone if required. This regimen was introduced given the unique short‐onset and long‐offset of the medication to provide more effective and longer‐lasting pain relief. This also has the additional benefit of reducing total opioid dose by reducing the amount of immediate‐release as required opioid medication. The patient developed a fever day 1 post‐op, and a septic screen was performed. The patient was managed with IV cefazolin however required vasopressor support for hemodynamic compromise. The patient returned for second‐stage surgery day 2 post first‐stage operation, consisting of resection of residual mucinous neoplasm from the left pelvic sidewall, right pelvic sidewall, posteromedial right thigh, distal pre‐sacral region, and through the left anterior table of the distal sacrum (S4) into the left gluteus maximus. HIPEC was not performed due to concerns about sepsis in ICU. This was followed immediately by a left pedicled anterolateral thigh flap reconstruction to the perineal defect. The patient returned to ICU for routine care. Postoperative analgesia consisted of paracetamol 1 g six hourly, fentanyl infusion (200 mcg/h), ketamine infusion (12 mg/h), epidural (ropivacaine 0.2%), and IV methadone (5 mg BD). The following day, the epidural was removed, and fentanyl infusion was transitioned to oxycodone patient‐controlled analgesia (PCA), and ketamine was increased to 16 mg/h with no changes to methadone and paracetamol dosing. The patient experienced episodes of breakthrough pain which was not relieved PCA and ketamine alone, with methadone dosing being reported by the patient to subjectively improve her pain including improved comfort when resting and with movement and reduced somatic symptoms of pain such as anxiety and distress. On the third day following the second‐stage operation, the patient was transferred from ICU to ward‐level care, continuing with PCA, ketamine, and IV methadone dosing as per the day prior with good analgesic effect. Upon arrival to the ward, the patient experienced breakthrough pelvic pain associated with distress. The patient's pain was not relieved with the baseline PCA and ketamine regimen. It was noted that the patient had missed her evening methadone dose due to ICU to ward transfer, and only upon receiving the methadone dose did her pain improve, indicating the effectiveness of methadone in supporting the patient's pain, physical distress, and discomfort in addition to her baseline analgesia. The patient underwent routine care on the ward with a plan to taper off analgesia back to the baseline pre‐admission regimen.

## 3 DISCUSSION AND LITERATURE REVIEW

Opioid medications are used as main‐stay therapy for chronic cancer and non‐cancer pain.^1^, ^8^ Methadone is one particular drug in this family that carries high potential in this treatment domain but remains underutilized due to its complex pharmacology and uncertainty around conversions to and from other opioids.^8^, ^9^ Additional barriers to its wider use in the postoperative space include unfamiliarity with the medication, government regulation, and to some extent, the stigma surrounding its use in both patients and clinicians alike.

Despite these sentiments, mechanistically, methadone exhibits many desirable properties that may prompt its use in postoperative patients with complex analgesia requirements. Methadone is a highly potent mu‐opioid receptor agonist with affinity for central and peripheral kappa‐ and delta‐opioid receptors; it also exhibits NMDA receptor antagonism and is a presynaptic blocker of serotonin and noradrenaline.^10^ Additionally, it exhibits high (albeit variable) oral bioavailability (70%–80%), is highly lipophilic, displays a high volume of distribution and protein binding, and has a long dose‐dependent elimination half‐life (approximately 24 h, ranging up to 90 h).^1^, ^8^, ^9^, ^11^ In this way, methadone provides potent analgesia which is bolstered by a long half‐life that mimics the duration of action of extended‐release opioids while simultaneously conferring neuropathic pain relief through non‐competitive NMDA antagonism. Methadone also modulates pain stimulus propagation to limit the development of hyperalgesia and mitigate opioid tolerance.^5^, ^12^ Renal and hepatic impairment do not affect methadone clearance with about 50% of methadone and its metabolites excreted by the intestines and 50% excreted unchanged by the kidneys.^13^ However useful, these pharmacologic properties can also limit its utility. Specifically, the lipophilicity of methadone causes tissue accumulation with repeated doses and is compounded by the high volume of distribution promoting a tendency toward rapid dose escalation and a propensity for overdose in the titration period prior to the drug reaching steady state.^1^ In the absence of judicious monitoring in the postoperative period, this may predispose patients to a higher risk of narcosis which is less likely for other standard opioid analgesia regimens currently in use. Moreover, there is an enormous interpatient variability in methadone metabolism that can be attributed to genetic polymorphism of the hepatic cytochrome P450 (CYP) genes.^1^, ^8^, ^9^ Therefore, clinicians must also consider CYP enzyme‐inducer and enzyme‐inhibitor interactions that can also impact metabolism and thereby serum levels.^1^ From a clinical perspective, routine measurement of CYP biomarkers is not a standard or practice, and therefore, identification of patients that may be more or less sensitive to methadone is a further barrier to consider with respect to its implementation.

The mixed mechanism of action and complex pharmacology of methadone allow it to occupy a unique place in therapy for hard‐to‐treat pain syndromes which is highly relevant for cancer patients who often experience chronic pain secondary to the burden of their condition. However, methadone is often relegated to last‐line therapy or therapy reserved only for specialists in the field due to an overall unfamiliarity with the drug and a lack of consensus opinion on dose conversions from comparator opioids such as morphine.^1^ Due to this, many institutions do not consider methadone in the entirety of their options for complex analgesia regimens, which poses another barrier to methadone implementation due to inexperience and scope of competency. In the literature, the morphine‐to‐methadone ratio is variable and is dose‐dependent due to the complex pharmacologic properties of methadone ranging from 3:1 to 4:1 (morphine to methadone) up to 20:1 for higher doses or continuous infusions.^1^, ^12^ Furthermore, conversion ratios from other opiates are poorly defined with inconsistent interpatient variability described in the literature.^1^, ^14^, ^15^ Difficult conversions are not only a theme when switching to methadone from other opioids but are also apparent when switching between routes of administration of methadone with inconsistent recommendations in the literature suggesting the conversion ratio of oral methadone to IV methadone as anywhere from 2:1 to 1:1 with a vague caveat that cautions higher doses require a more conservative ratio.^1^, ^9^ This becomes relevant when tapering patients off methadone, with poorly characterized evidence as to best‐practice ways to approach this. For clinicians and stakeholders involved in guideline and policy development, rigorous research to characterize dose conversions and step‐down conversion processes is highly important to drive the wider and safe implementation of methadone in postoperative pain management.

The side effect profile of methadone is another consideration. The primary concern is that of the implications on respiratory depression on the background of the medications' long, unpredictable, and dose‐dependent half‐life.^9^ The apparent effects of methadone can be attributed to its racemic nature with the R‐enantiomer responsible for both beneficial analgesic effects but also undesirable side effects, and the S‐enantiomer causing the antitussive and cardiac conduction effects.^13^ Due to the increasing alarm around risk of cardiac QT interval prolongation and thus Torsades de Pointes (TdP), there has been some debate in the literature as to the benefits and drawbacks of prescribing the racemic mixture over the R‐enantiomer of methadone.^13^, ^16^ The Therapeutic Goods Administration (TGA) is a government authority responsible for assessing and monitoring products defined as “therapeutic goods” in Australia. The TGA has approved the use of oral and IV methadone as a racemate (at the time of writing only products branded Physeptone or Methadone Syrup from Aspen Pharmacare and Sigma Pharmaceuticals, respectively, or Biodene Forte from Biomed Australia are licensed for use in Australia) for opioid replacement therapy or treatment of chronic or palliative pain where pain is known to be opioid responsive and where other treatment options have failed or are contraindicated.^17^ Similarly, these products have strict access criteria under the Pharmaceutical Benefits Scheme (PBS) wherein which cost of pharmaceuticals is subsidized by the Australian Government on the proviso that certain criteria are met. For methadone, this is further restricted in that the prescriber must obtain an authority approval (a telephone or written approval from Services Australia), and although this process has now been streamlined, the access criteria remain strictly for opioid dependence (oral liquid for opioid replacement therapy) and for the treatment of severe disabling pain in non‐opioid naïve patients undergoing palliative care.^18^, ^19^

Extending from this, clinicians and patients also need to consider the social implications of instituting methadone‐based regimens for postoperative analgesia. The stigma around methadone and its links to opioid addiction (including illicit substances such as heroin) were highlighted as a barrier clearly in this case and also reflected in the literature.^20^, ^21^, ^22^ Despite these sentiments, it is clear from community opioid rehabilitation programs that supervised methadone is safe and well‐tolerated. Therefore, stakeholders involved in guideline or policy development for methadone institution, clinicians, pharmacists and all healthcare workers involved in the procurement, preparation, and delivery of the drug should equally consider engaging with educational programs aimed in promoting de‐stigmatization and safe use of methadone in the postoperative environment.

In specialist settings, such as that of a tertiary hospital, the use of methadone is not limited to treatment of opioid dependence or chronic pain and has a growing body of evidence that supports intraoperative doses in a variety of surgeries to lessen postoperative pain scores and thus minimize postoperative opioid requirements.^2^, ^9^, ^12^, ^23^ However, there is a paucity of evidence that supports the use of methadone as breakthrough analgesia in the first instance. Specifically, the Australian and New Zealand College of Anesthetists and Faculty of Pain Medicine (ANZCA & FCM) have discouraged breakthrough use of methadone, which is considered a slow‐release opioid due to lack of evidence‐based consensus data on its' feasibility and dose titration.^8^ Of the various studies that have examined IV methadone, almost all have suggested single intraoperative doses may produce more effective pain control and may lower postoperative opioid requirements without more adverse effects when compared to comparator opioids such as morphine.^5^, ^24^, ^25^ Anecdotally, outside of the operating room and post‐anesthetics recovery units, orders for breakthrough doses of methadone (either IV, subcutaneous or intramuscular) are a rarity, and subsequently, administration can be a cause for concern. Furthermore, this hesitancy extends to nursing and pharmacy staff who may also be inexperienced with the acquisition, preparation, and delivery of methadone in the clinical context. Moreover, there remain significant limitations with respect to the clinical trials which evaluate the use of perioperative methadone. Murphy et al.^9^ highlight in a review of this topic key issues with methodological rigor associated with poorly powered studies of small sample sizes leading to subsequent bias in overestimation of effect size and false positive results of effect. To best streamline the safe adoption of methadone in pain regimens, it is clear further research examining the effect and tolerance of this medication with judicious attention to dose conversion, uptitration, and downtitration regimens is required. Additionally, exploring the efficacy of methadone in specific surgical settings, such as with high‐risk patient populations (elderly, morbidly obese, and those with a number of significant multisystem comorbidities), is imperative to ensure that the use of this drug maintains its safety and tolerance in the diverse range of patients whom present with cancer and chronic or complex pain requirements.^9^

## 4 CONCLUSION

At this point in time, there is insufficient robust evidence to suggest the evidence‐based incorporation of IV methadone into the regimen of postoperative care of patients, regardless of whether the patient has complex analgesia requirements or not. From a clinical perspective, stigma regarding the use of methadone should not, in the context of judicious pain management, be a significant barrier to the use of methadone. However, inexperience and poor characterization of methadone titration and effect pose an important barrier toward implementation. Despite this, evidence suggests that methadone may offer favorable analgesic effects in the postoperative period, with lower pain scores, improved patient satisfaction, and lower opioid consumption.^7^ As surgeons and perioperative clinicians adopt enhanced recovery after surgery (ERAS) protocols, the focus on efficiently and efficaciously reducing postoperative opioid consumption prompts consideration into methadone as a potential therapeutic.

## AUTHOR CONTRIBUTIONS

**Khang Duy Ricky Le:** Conceptualization; data curation; formal analysis; investigation; methodology; project administration; supervision; validation; visualization; writing – original draft; writing – review and editing. **Jean Hua:** Conceptualization; data curation; formal analysis; investigation; methodology; validation; writing – original draft; writing – review and editing.

## FUNDING INFORMATION

There are no conflicts of interest or disclaimers.

## CONFLICT OF INTEREST STATEMENT

There was financial and grant support.

## ETHICS STATEMENT

The case report generation process was discussed with the Peter MacCallum Cancer Centre ethics and governance team. No formal ethics approval was required following the discussions.

## CONSENT

The patient provided informed consent that was written and signed for generation and publication of this manuscript using their de‐identified medical information.

## Data Availability

The data that support the findings of this study are available from the corresponding author upon reasonable request.

## References

[1] Shaiova L , Berger A , Blinderman CD , et al. Consensus guideline on parenteral methadone use in pain and palliative care. Palliat Support Care. 2008;6(2):165‐176. 18501052 10.1017/S1478951508000254

[2] Kharasch ED . Intraoperative methadone: rediscovery, reappraisal, and reinvigoration? Anesth Analg. 2011;112(1):13‐16. 21173206 10.1213/ANE.0b013e3181fec9a3 PMC3689220

[3] Bravo L , Llorca‐Torralba M , Berrocoso E , Micó JA . Monoamines as drug targets in chronic pain: focusing on neuropathic pain. Front Neurosci. 2019;13:1268. 31942167 10.3389/fnins.2019.01268 PMC6951279

[4] Latremoliere A , Woolf CJ . Central sensitization: a generator of pain hypersensitivity by central neural plasticity. J Pain. 2009;10(9):895‐926. 19712899 10.1016/j.jpain.2009.06.012 PMC2750819

[5] Mercadante S . Intravenous methadone for perioperative and chronic cancer pain: a review of the literature. Drugs. 2023;83(10):865‐871. 37308798 10.1007/s40265-023-01876-7

[6] Mercadante S , Adile C , Ferrera P , et al. Methadone as first‐line opioid for the management of cancer pain. Oncologist. 2022;27(4):323‐327. 35380722 10.1093/oncolo/oyab081 PMC8982366

[7] Machado FC , Vieira JE , de Orange FA , Ashmawi HA . Intraoperative methadone reduces pain and opioid consumption in acute postoperative pain: a systematic review and meta‐analysis. Anesth Analg. 2019;129(6):1723‐1732. 31743194 10.1213/ANE.0000000000004404

[8] Schug SA , Palmer GM , Scott DA , Alcock M , Halliwell R , Mott J . Acute pain management: scientific evidence. Med J Aust. 2020;204:315‐317. 10.5694/mja16.00133 27125806

[9] Murphy GS , Szokol JW . Intraoperative methadone in surgical patients: a review of clinical investigations. Anesthesiology. 2019;131(3):678‐692. 31094758 10.1097/ALN.0000000000002755

[10] Edmonds KP , Saunders IM , Willeford A , Ajayi TA , Atayee RS . Emerging challenges to the safe and effective use of methadone for cancer‐related pain in paediatric and adult patient populations. Drugs. 2020;80:115‐130. 31820362 10.1007/s40265-019-01234-6

[11] Ferrari A , Coccia CPR , Bertolini A , Sternieri E . Methadone—metabolism, pharmacokinetics and interactions. Pharmacol Res. 2004;50(6):551‐559. 15501692 10.1016/j.phrs.2004.05.002

[12] D'Souza RS , Gurrieri C , Johnson RL , Warner N , Wittwer E . Intraoperative methadone administration and postoperative pain control: a systematic review and meta‐analysis. Pain. 2020;161(2):237‐243. 31613867 10.1097/j.pain.0000000000001717

[13] Royal Pharmaceutical Society . Methadone [Internet]. Palliative Care Formulary [MedicinesComplete]. Royal Pharmaceutical Societ. 2021.

[14] Manfredi PL , Borsook D , Chandler SW , Payne R . Intravenous methadone for cancer pain unrelieved by morphine and hydromorphone: clinical observations. Pain. 1997;70(1):99‐101. 9106815 10.1016/s0304-3959(96)03313-1

[15] Auret K , Goucke CR , Ilett KF , Page‐Sharp M , Boyd F , Oh TE . Pharmacokinetics and pharmacodynamics of methadone enantiomers in hospice patients with cancer pain. Ther Drug Monit. 2006;28(3):359‐366. 16778720 10.1097/01.ftd.0000211827.03726.e4

[16] Gaertner J , Voltz R , Ostgathe C . Methadone: a closer look at the controversy. J Pain Symptom Manage. 2008;36(2):e4‐e7. 10.1016/j.jpainsymman.2008.04.007 18655953

[17] The Therapeutic Goods Administration . Methadone: Australian Government: Department of Health and Aged Care. 2023. Accessed November 17, 2023. https://www.tga.gov.au/search?keywords=methadone&submit=Search

[18] Pharmaceutical Benefits Scheme . Methadone: Australian Government: Department of Health and Aged Care. 2023. Accessed November 17, 2023. https://www.pbs.gov.au/pbs/search?term=methadone

[19] Pharmaceutical Benefits Scheme . Fact Sheet: Streamlined Authorities: Australian Government: Department of Health and Aged Care. 2020. Accessed November 17, 2023.https://www.pbs.gov.au/info/publication/factsheets/shared/fact‐sheet‐streamlined‐authorities

[20] Shah S , Diwan S . Methadone: does stigma play a role as a barrier to treatment of chronic pain? Pain Physician. 2010;13(3):289‐293. 20495594

[21] Anstice S , Strike CJ , Brands B . Supervised methadone consumption: client issues and stigma. Subst Use Misuse. 2009;44(6):794‐808. 19444722 10.1080/10826080802483936

[22] Conner KO , Rosen D . “You're nothing but a junkie”: multiple experiences of stigma in an aging methadone maintenance population. J Soc Work Pract Addict. 2008;8(2):244‐264.

[23] Berde CB , Beyer JE , Bournaki M‐C , Levin CR , Sethna NF . Comparison of morphine and methadone for prevention of postoperative pain in 3‐to 7‐year‐old children. J Pediatr. 1991;119(1):136‐141. 2066846 10.1016/s0022-3476(05)81054-6

[24] Gourlay G , Willis RJ , Lamberty J . A double‐blind comparison of the efficacy of methadone and morphine in postoperative pain control. Anesthesiology. 1986;64(3):322‐327. 3954126 10.1097/00000542-198603000-00004

[25] Richlin DM , Reuben SS . Postoperative pain control with methadone following lower abdominal surgery. J Clin Anesth. 1991;3(2):112‐116. 2039637 10.1016/0952-8180(91)90007-a

